# Supplementary material for: Fingerprinting of skin cells by live cell Raman spectroscopy reveals melanoma cell heterogeneity and cell‐type‐specific responses to UVR
Source: Exp Dermatol. 2022 Jun 27;31(10):1543–53. doi: 10.1111/exd.14625 (PMC9796253; doi:10.1111/exd.14625)
Supplement: Supplementary file 1 — Figure S1. Live Raman spectroscopy can discriminate between cell types and differentiation status. Raman spectra (n = 9 for each cell type) were acquired from melanocytes (melan‐a), melanoblasts (MelbA), melanoma cells (B16F10) and keratinocytes (COCA). Cells were grown on CaF2 disks in phenol red free medium prior to acquisition at 785 nm. A principal component analysis (PCA) was then conducted. (a) Bright field images of the cell types used. (b) The mean Raman spectra (offset for clarity) for each cell type differed qualitatively. (c) A scatter plot of the principal component vectors PC1 (98.27%) and PC2 (1.05%) showing tight grouping of melanoblasts, melanocytes and keratinocytes with a more heterogeneous melanoma population. (d) PCA loadings of the PC1 and PC2 vectors ‐ the ten principal Raman peaks are labelled with arrows on (b). The y‐axis separation of the PCA loading plots in b and d is for visual purposes only. Scale bar in A = 200 μm. Numbers in (b): 1 = Lipid, 2 = Phenylalanine, 3 = Phospholipids/Tryptophan, 4 = Lipid, 5 = DOPA, 6 = β‐carotene, 7 = Keratin, 8 = Melanin, 9 = Tyrosine, 10 = Amide I. Figure S2. Cell viability post Raman spectral acquisition. melan‐a, melbA, B16F10 and COCA cells were grown on CaF2 disks in duplicate in phenol red free medium for 24 h before irradiation with 100 KJ/m2 UVA, 1000 J/m2 UVA and UVB or 100 J/m2 UVB before spectra acquired from one disk at 1, 3, 6, 16 and 24 h post radiation as well as from an untreated control. The other disk was maintained under normal culture conditions as a control. Cell viability was measured using Cell Titer Glo. (A) UVA, (B) UVB and (C) UVA and UVB. Data represented as mean ± 95% CI, N = 18. Figure S3. Raman spectra of synthetic melanin and DOPA. The spectra of melanin and DOPA was acquired using a 785 nm laser at a spectral range of 600–1700 cm−1. Spectra was collected at 2 seconds with 9 accumulations (18 s total) at 100% laser power. Data collected was baseline corrected and smoothed usi [file EXD-31-1543-s001.pdf]

SUPPLEMENTARY MATERIAL:

SUPPLEMENTARY TABLES:

Table S1: Summary of Raman spectral peaks.

**Peak # (Fig. 1E)**	**Peak (cm^{-1})**	**Assignment**	**Function**	**Reference**
1	972	Lipid		Movasaghi et al. 2015
2	1004	Phenylalanine	Melanin biosynthesis	Morita, Takanezawa et al. 2013, Talari, Movasaghi et al. 2015
3	1123	Phospholipids/ Tryptophan		Talari, Movasaghi et al. 2015
4	1300	Lipid		Talari, Movasaghi et al. 2015
5	1333	DOPA	Melanin biosynthesis	Supplementary figure 2
6	1396	β-carotene		Talari, Movasaghi et al. 2015
7	1450	Keratin		Feng, Moy et al. 2017
8	1580	Melanin	Melanin biosynthesis	Huang, Lui et al. 2004, Morita, Takanezawa et al. 2013, Moncada, Castillo-Martinez et al. 2016 and supplementary figure 2
9	1614	Tyrosine	Melanin biosynthesis	Talari, Movasaghi et al. 2015
10	1650	Amide I		Talari, Movasaghi et al. 2015

Table S2: Oligonucleotides used for RT-qPCR analysis.

Gene	Forward Primer (5’>3’)	Reverse Primer (5’>3’)
Dct	CATGGTCCCCTTCTTCCCAC	GAAAAGCCAGCAACCCCAAG
Mitf	CCCAGGTATGAACACGCACT	GCTGGACAGGAGTTGCTGAT
Tyr	CCTGAGGGACCACTATTACG	CTATCCCTGTGAGTGGACTG
Tyrp1	GTTCAATGGCCAGGTCAGGA	CAGTGAGGAGAGGCTGGTTG

SUPPLEMENTARY FIGURES:

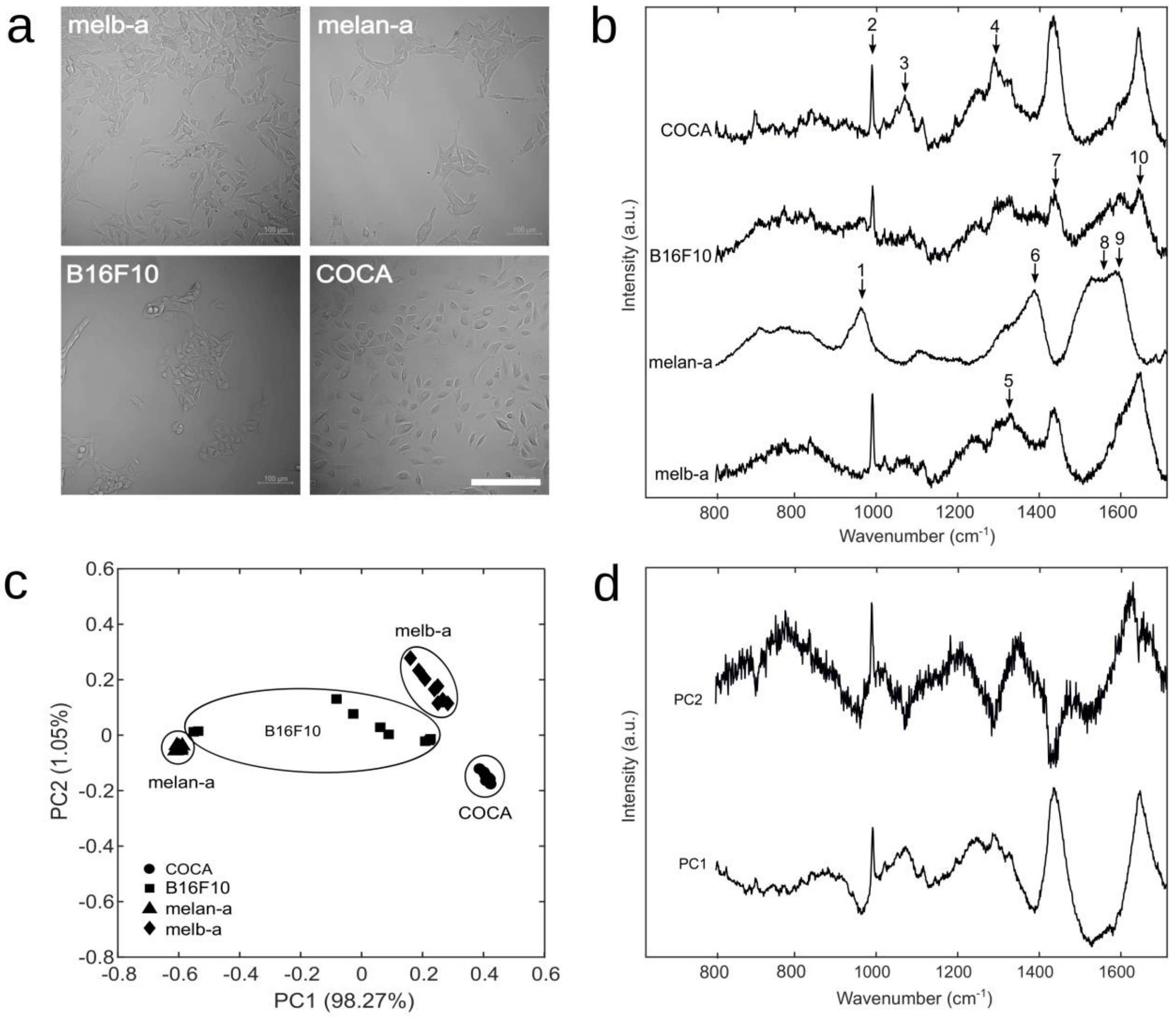

Figure S1: Live Raman spectroscopy can discriminate between cell types and differentiation status. Raman spectra (n = 9 for each cell type) were acquired from melanocytes **(**melan-a), melanoblasts (melb-a), melanoma cells (B16F10) and keratinocytes (COCA). Cells were grown on CaF_2 disks in phenol red free medium prior to acquisition at 785nm. A principal component analysis (PCA) was then conducted. (a) Bright field images of the cell types used. (b) The mean Raman spectra (offset for clarity) for each cell type differed qualitatively. (c) A scatter plot of the principal component vectors PC1 (98.27%) and PC2 (1.05%) showing tight grouping of melanoblasts, melanocytes and keratinocytes with a more heterogeneous melanoma population. (d) PCA loadings of the PC1 and PC2 vectors - the ten

principal Raman peaks are labelled with arrows on (b). The y-axis separation of the PCA loading plots in b and d is for visual purposes only. Scale bar in A = 200 µm. Numbers in (b): 1 = Lipid, 2 = Phenylalanine, 3 = Phospholipids/Tryptophan, 4 = Lipid, 5 = DOPA, 6 = β-carotene, 7 = Keratin, 8 = Melanin, 9 = Tyrosine, 10 = Amide I.

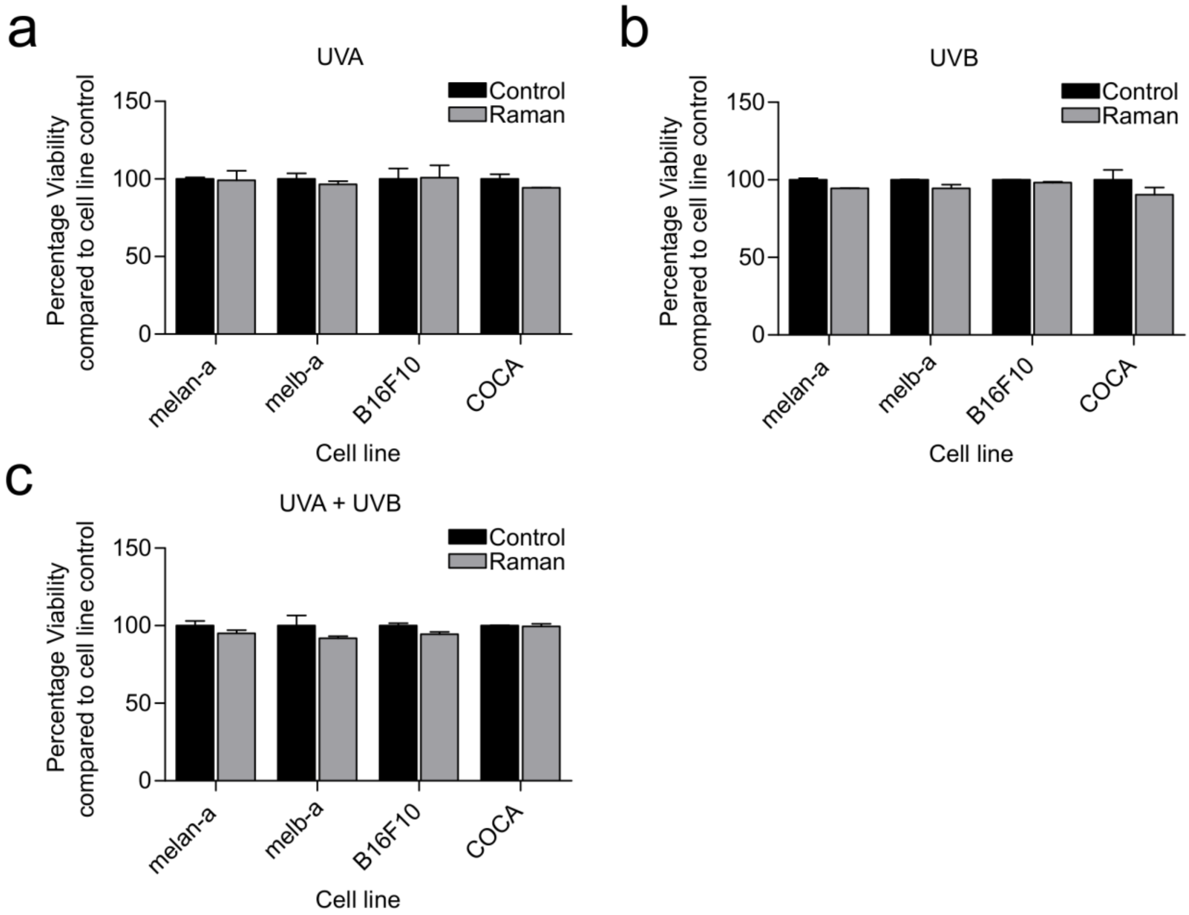

<u>Figure S2. Cell viability post Raman spectral acquisition.</u> melan-a, melb-a, B16F10 and COCA cells were grown on CaF_2 disks in duplicate in phenol red free medium for 24 hours before irradiation with 100KJ/m^2 UVA, 1000J/m^2 UVA and UVB or 100J/m^2 UVB before spectra acquired from one disk at 1, 3, 6, 16 and 24 hours post radiation as well as from an untreated control. The other disk was maintained under normal culture conditions as a control. Cell viability was measured using Cell Titer Glo. (A) UVA, (B) UVB and (C) UVA and UVB. Data represented as mean ± 95% CI, N=18.

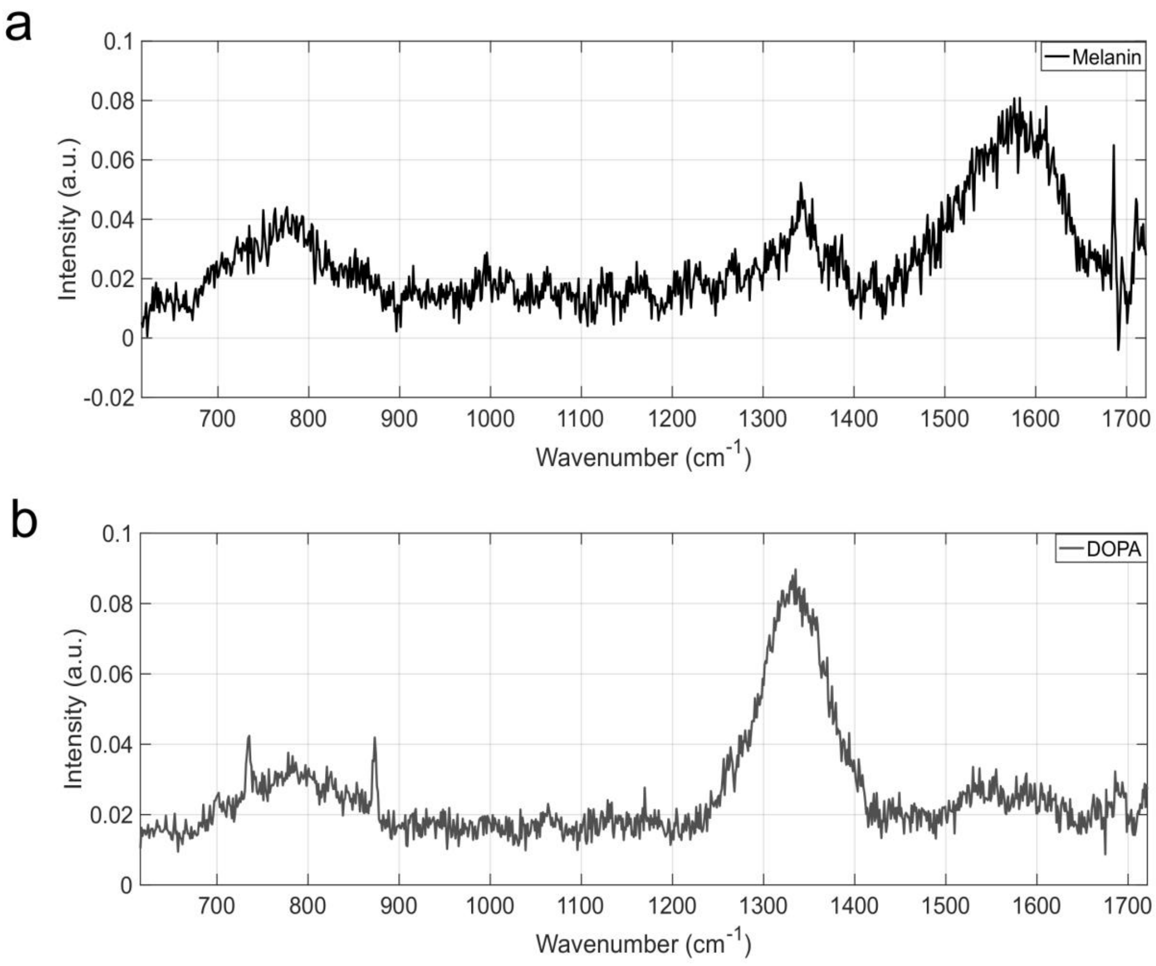

Figure S3. Raman spectra of synthetic melanin and DOPA. The spectra of melanin and DOPA was acquired using a 785nm laser at a spectral range of 600-1700cm^{-1}. Spectra was collected at 2 seconds with 9 accumulations (18 seconds total) at 100% laser power. Data collected was baseline corrected and smoothed using P=0.001, λ=105 before vector normalising.

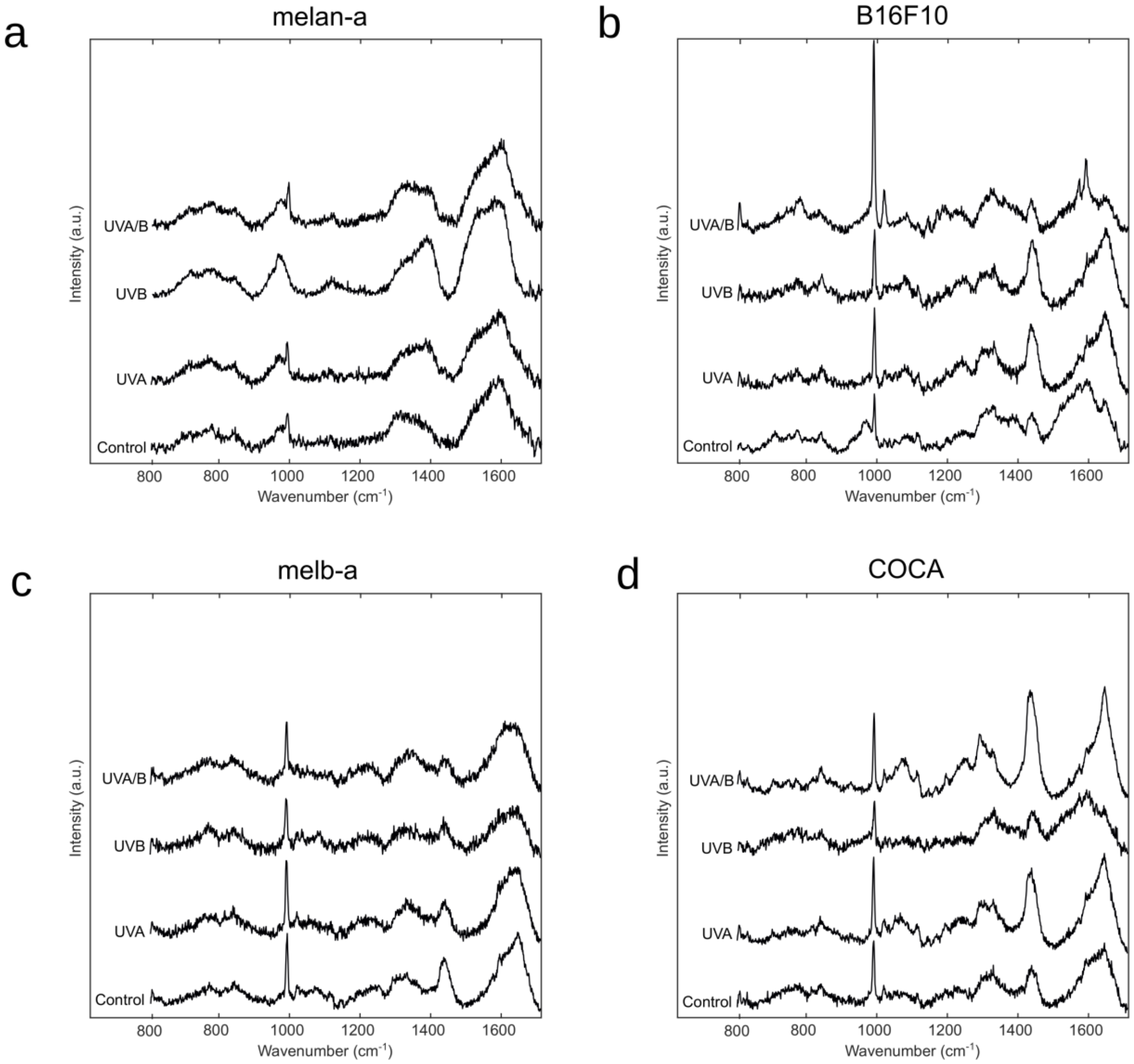

<u>Figure S4. Raman spectra of UVR treated cells.</u> melan-a, melb-a, B16F10 and COCA cells were grown on CaF_2 disks and irradiated with 100KJ/m^2 UVA, 1000J/m^2 UVA and UVB or 100J/m^2 UVB before Raman spectra acquired using a 785nm laser at a spectral range of 600-1700cm^{-1}, 100% laser power 2 seconds with 9 accumulations (18 seconds total) at 24 hours post irradiation and compare to an untreated control. Data collected was baseline corrected and smoothed using P=0.001, λ=105 before vector normalising. Raman spectra for each cell line plotted as class mean (n=18) (A) melan-a, (B) B16F10, (C) melb-a, (D) COCA. The y-axis separation of the spectra in plots a-d is for visual purposes only.
